# Plasma MCP-1 and changes on cognitive function in community-dwelling older adults

**DOI:** 10.1186/s13195-021-00940-2

**Published:** 2022-01-07

**Authors:** Juan Luis Sanchez-Sanchez, Kelly V. Giudici, Sophie Guyonnet, Julien Delrieu, Yan Li, Randall J. Bateman, Angelo Parini, Bruno Vellas, Philipe de Souto Barreto, for the MAPT/DSA Group, Bruno Vellas, Sophie Guyonnet, Isabelle Carrié, Lauréane Brigitte, Catherine Faisant, Françoise Lala, Julien Delrieu, Hélène Villars, Emeline Combrouze, Carole Badufle, Audrey Zueras, Sandrine Andrieu, Christelle Cantet, Christophe Morin, Gabor Abellan Van Kan, Charlotte Dupuy, Yves Rolland, Céline Caillaud, Pierre-Jean Ousset, Françoise Lala, Sherry Willis, Sylvie Belleville, Brigitte Gilbert, Francine Fontaine, Jean-François Dartigues, Isabelle Marcet, Fleur Delva, Alexandra Foubert, Sandrine Cerda, Marie-Noëlle-Cuffi, Corinne Costes, Olivier Rouaud, Patrick Manckoundia, Valérie Quipourt, Sophie Marilier, Evelyne Franon, Lawrence Bories, Marie-Laure Pader, Marie-France Basset, Bruno Lapoujade, Valérie Faure, Michael Li Yung Tong, Christine Malick-Loiseau, Evelyne Cazaban-Campistron, Françoise Desclaux, Colette Blatge, Thierry Dantoine, Cécile Laubarie-Mouret, Isabelle Saulnier, Jean-Pierre Clément, Marie-Agnès Picat, Laurence Bernard-Bourzeix, Stéphanie Willebois, Iléana Désormais, Noëlle Cardinaud, Marc Bonnefoy, Pierre Livet, Pascale Rebaudet, Claire Gédéon, Catherine Burdet, Flavien Terracol, Alain Pesce, Stéphanie Roth, Sylvie Chaillou, Sandrine Louchart, Kristel Sudres, Nicolas Lebrun, Nadège Barro-Belaygues, Jacques Touchon, Karim Bennys, Audrey Gabelle, Aurélia Romano, Lynda Touati, Cécilia Marelli, Cécile Pays, Philippe Robert, Franck Le Duff, Claire Gervais, Sébastien Gonfrier, Yannick Gasnier, Serge Bordes, Danièle Begorre, Christian Carpuat, Khaled Khales, Jean-François Lefebvre, Samira Misbah El Idrissi, Pierre Skolil, Jean-Pierre Salles, Carole Dufouil, Stéphane Lehéricy, Marie Chupin, Jean-François Mangin, Ali Bouhayia, Michèle Allard, Frédéric Ricolfi, Dominique Dubois, Marie Paule Bonceour Martel, François Cotton, Alain Bonafé, Stéphane Chanalet, Françoise Hugon, Fabrice Bonneville, Christophe Cognard, François Chollet, Pierre Payoux, Thierry Voisin, Sophie Peiffer, Anne Hitzel, Michel Zanca, Jacques Monteil, Jacques Darcourt, Laurent Molinier, Hélène Derumeaux, Nadège Costa, Bertrand Perret, Claire Vinel, Sylvie Caspar-Bauguil, Pascale Olivier-Abbal, Nicola Coley

**Affiliations:** 1 Gérontopôle de Toulouse, Institut du Vieillissement, Centre Hospitalier-Universitaire de Toulouse, 37 allées Jules Guesde, 31000 Toulouse, France; 2 Faculty of Sport Sciences, Universidad Europea de Madrid, Villaviciosa de Odón, 28670 Madrid, Spain; 3 CERPOP, Inserm 1295, Université de Toulouse, UPS, Toulouse, France; 4 Department of Neurology, Washington University in St. Louis, St. Louis, MO USA; 5 Division of Biostatistics, Washington University in St. Louis, St. Louis, MO USA; 6 Knight Alzheimer’s Disease Research Center, Washington University in St. Louis, St. Louis, MO USA; 7 Hope Center for Neurological Disorders, Washington University in St. Louis, St. Louis, MO USA; 8 Institut des Maladies Métaboliques et Cardiovasculaires, Inserm/Université Paul Sabatier UMR 1048 – I2MC 1, Toulouse, France

**Keywords:** MCP-1, Older adults, Alzheimer’s disease, Cognitive function, Episodic memory

## Abstract

**Background:**

Monocyte Chemoattractant Protein-1 (MCP-1), a glial-derived chemokine, mediates neuroinflammation and may regulate memory outcomes among older adults. We aimed to explore the associations of plasma MCP-1 levels (alone and in combination with β-amyloid deposition—Aβ_42/40_) with overall and domain-specific cognitive evolution among older adults.

**Methods:**

Secondary analyses including 1097 subjects (mean age = 75.3 years ± 4.4; 63.8% women) from the Multidomain Alzheimer Preventive Trial (MAPT). MCP-1 (higher is worse) and Aβ_42/40_ (lower is worse) were measured in plasma collected at year 1. MCP-1 in continuous and as a dichotomy (values in the highest quartile (MCP-1^+^)) were used, as well as a dichotomy of Aβ_42/40_. Outcomes were measured annually over 4 years and included the following: cognitive composite *z*-score (CCS), the Mini-Mental State Examination (MMSE), and Clinical Dementia Rating (CDR) sum of boxes (overall cognitive function); composite executive function *z*-score, composite attention *z*-score, Free and Cued Selective Reminding Test (FCSRT - memory).

**Results:**

Plasma MCP-1 as a continuous variable was associated with the worsening of episodic memory over 4 years of follow-up, specifically in measures of free and cued delayed recall. MCP-1^+^ was associated with worse evolution in the CCS (4-year between-group difference: *β* = −0.14, 95%CI = −0.26, −0.02) and the CDR sum of boxes (2-year: *β* = 0.19, 95%CI = 0.06, 0.32). In domain-specific analyses, MCP-1^+^ was associated with declines in the FCSRT delayed recall sub-domains. In the presence of low Aβ_42/40_, MCP-1^+^ was not associated with greater declines in cognitive functions. The interaction with continuous biomarker values *Aβ*_*42/40*_*× MCP-1 × time* was significant in models with CDR sum of boxes and FCSRT DTR as dependent variables.

**Conclusions:**

Baseline plasma MCP-1 levels were associated with longitudinal declines in overall cognitive and episodic memory performance in older adults over a 4-year follow-up. How plasma MCP-1 interacts with Aβ_42/40_ to determine cognitive decline at different stages of cognitive decline/dementia should be clarified by further research. The MCP-1 association on cognitive decline was strongest in those with amyloid plaques, as measured by blood plasma *Aβ*_*42/40.*_

**Supplementary Information:**

The online version contains supplementary material available at 10.1186/s13195-021-00940-2.

## Introduction

Declines in cognitive function during aging is one of the most important public health challenges of the coming decades [1]. Early identification of older adults at risk of cognitive decline through the use of accessible and reliable biomarkers may inform timely intervention [2]. In this context, blood-born analytes have gained attention because of their feasibility, potential widespread use [2–5], and their association with cognitive outcomes and dementia onset in samples of older adults [6–8], suggesting that they may be predictors of cognitive function decline [9].

Immune dysregulation, characterized by chronic and exacerbated glial polarization, contributes to cognitive decline by promoting neurodegeneration (synaptic loss and neuronal death) [10]. In such dysregulated state, microglial cells release several inflammatory molecules [3] that contribute to microglial and astrocytic polarization, initiating a self-perpetuating cycle [11, 12]. Among them, the chemokine monocyte chemo-attractant protein-1—MCP-1, also known as C-C motif ligand 2 [13, 14], stands out given its tight relation to neuroimmune dysfunction [15]. This chemokine is an important regulator of monocyte/lymphocyte migration and infiltration into CNS through its interaction with CC-chemokine receptor 2 [16].

Although animal- [17, 18] and human-based [19–24] studies have linked increased CSF and plasma levels of MCP-1 with functional and brain structural changes associated with cognitive decline in older adults, most previous studies were cross-sectional and included either healthy subjects or people with a dementia diagnosis. No study investigated early stages of cognitive decline, such as people with spontaneous memory complaints and those with mild cognitive impairment [25].

Therefore, the main objective of the present study is to investigate the associations between plasma MCP-1 alone and overall and domain-specific cognitive evolution among community-dwelling older adults. Secondarily, we aimed to explore its interaction with plasma β-amyloid.

## Methods

### Study design and population

This observational longitudinal analysis uses data from the Multidomain Alzheimer Preventive Trial (MAPT, ClinicalTrials.gov [NCT00672685]), a randomized, multicenter, placebo-controlled trial conducted with community-dwelling older adults in France and Monaco. Participants were allocated into 4 groups, either receiving ω-3 polyunsaturated fatty acid (PUFA) supplementation, a multidomain intervention (based on cognitive training, nutritional counseling, and physical activity advice), both, or placebo. The intervention lasted for 3 years and was followed by an additional 2-year observational phase. Recruitment of participants started in May 2008 and ended in February 2011. Follow-up ended in April 2016.

Detailed description of the MAPT study can be found elsewhere [26, 27]. In summary, eligibility criteria comprised the following: age 70 years or older; not presenting major neurocognitive disorders, Mini-Mental State Examination [MMSE] score, ≥ 24; presenting at least 1 of the following: spontaneous memory concern, inability to perform 1 instrumental activity of daily living (ADL), or slow usual-pace walking speed (< 0.8 m/s). Participants were not included if they declared the use of ω-3 PUFA supplements during the 6 months before inclusion.

The population of the present study was composed of 1097 subjects with data on plasma MCP-1; among them, 429 individuals also had information on plasma β-amyloid. The present study followed the Strengthening the Reporting of Observational Studies in Epidemiology (STROBE) guideline [28].

### MCP-1 and Aβ42/40 status

Plasma MCP-1 levels were measured at the MAPT 1-year visit using the fully automated immunoassay platform, ELLA (ProteinSimple/Bio-Techne, San Jose, CA, USA). MCP-1 levels were displayed as pg/mL. For analytical purposes, in the absence of cut-points to define plasma MCP-1 abnormal values, we used the quartiles in MCP-1 levels to define high values of the MCP-1 (MCP-1^+^; Q4 > 251 pg/mL) and MCP-1^−^ (≤ Q4) groups.

We used the plasma Aβ42/40 ratio as a marker of β-amyloid deposition at 1-year visit [29]. Plasma samples were spiked with a known quantity of 15N-Aβ42 and 15N-Aβ40 for use as analytical internal standards. A full description of the immunoprecipitation methods applied has been previously described [30]. Briefly, Aβ42 and Aβ40 isoforms were simultaneously immunoprecipitated from 0.45 mL of plasma via a monoclonal anti-Aβ middomain antibody (HJ5.1, anti-Aβ13-28) conjugated to M-270 Epoxy Dynabeads (Invitrogen). Protein digestion into peptides was done using LysN endoprotease (Pierce). Liquid chromatography–mass spectrometry was performed as detailed elsewhere [30]. Plasma analyses were performed as targeted parallel reaction monitoring on an Orbitrap Fusion Lumos Tribrid mass spectrometer (Thermo Fisher) interfaced with an M-class nanoAcquity chromatography system (Waters). The precursor and product ion pairs used for analysis of Aβ isoforms were chosen as previously detailed [31, 32]. Derived integrated peak areas were analyzed using the Skyline software package [33]. Aβ_42_ and Aβ_40_ quantities (in pg/mL) were calculated by integrated peak area ratios to known concentrations of the internal standards. The plasma Aβ_42/40_ ratio was then calculated by dividing Aβ_42_ by Aβ_40_ and its normalized values were used to classify Aβ status (determined by Youden index as low (Aβ_42/40_^+^ ≤ 0.107) and normal (Aβ_42/40_^−^ > 0.107), using β-amyloid load assessed by Positron Emission Tomography as the reference standard).

Specification of the assays used (limits of detection, coefficient of variation and linearity) are displayed in Additional file 1.

### Outcome measures

Outcomes were assessed in the same visit in which MCP-1 and Aβ_42/40_ were measured (data collected before MCP-1 measurement were not used); outcomes were prospectively evaluated annually for 4 years. Overall cognitive performance was assessed using the following: a composite cognitive score (CCS) [27] based on four tests (the 10 orientation items of the MMSE, the Digit Symbol Substitution Test (DSST), free and total recall of the Free and Cued Selective Reminding Test (FCSRT), and the Category Naming Test), the MMSE score [34], and the Clinical Dementia Rating (CDR) sum of boxes [35].

Specific cognitive domains evaluated were as follows: (a) episodic memory (from the FCSRT) [36], (b) executive function (based on the composite of the *z*-scores of the Controlled Oral Word Association Test [37], the CNT [38], and the Trail Making Test-Part B [39]), and (c) attention (based on the composite of the *z*-scores of the DSST [40] and the Trail Making Test-Part A [39]). All these instruments were administered following standard procedures.

### Potential confounders

Potential confounders consisted of age, sex, body mass index (BMI; kg/m^2^), MAPT group allocation, CDR status at baseline (CDR score 0; 0.5, or ≥ 1), 15-item Geriatric Depression Scale (GDS) [41], and apolipoprotein E (ApoE) ε4 genotype (carrier of at least one allele vs non-carrier). All confounders were measured at the 1-year MAPT visit.

### Statistical analysis

Descriptive statistics (mean ± standard deviation or frequencies and percentages, as appropriate) were used for the characterization of the study population. Quantitative variables at baseline (the 1-year visit where plasma MCP-1 and Aβ were measured) were compared according to MCP-1 status by Student’s *t* tests, and categorical variables were compared using the *χ*^2^ tests.

Linear mixed-effects (LME) regression analyses (with random intercept and random slope for each participant) were performed to determine associations between baseline plasma MCP-1 (either continuous or dichotomous) and the annual rate of changes in the outcome measures. The LME models included the fixed effects of baseline plasma MCP-1, time, their interaction, and potential confounders (model 1: all the covariates described; model 2: all confounders except ApoE ε4 genotype) [42]. The rationale for removing ApoE ε4 genotype in a second model was the substantial number of subjects with missing information (110 out of 1097, 10% of the sample). Using similar adjusted models, we further investigated the joint associations of baseline MCP-1 (as a continuous variable) and Aβ_42/40_ status with the rate of change of cognitive outcomes by including a three-way interaction (*Aβ*_*42/40*_*× MCP-1 × time*) in the fixed effects, in addition to the main effects and the two-way interactions between these variables. For the categorical approach, we defined four groups: Aβ_42/40_^−^/MCP-1^−^ (reference category), Aβ_42/40_^−^/MCP-1^+^, Aβ_42/40_^+^/MCP-1^−^, Aβ_42/40_^+^/MCP-1^+^). In the analyses of the CDR sum of boxes, CDR status at baseline was not included as a covariate.

In post-hoc analyses, we investigated wether MCP-1 associated with hippocampal volume changes (in cm^3^, assessed by magnetic resonance imaging—MRI) and β-amyloid deposition (cortical-to-cerebellar regional mean standardized uptake value ratio [SUVr] assessed by positron emission tomography—PET). A composite value computed as the mean of six predefined anatomically relevant cortical regions of interest (frontal, temporal, parietal, precuneus, anterior cingulate, and posterior cingulate [composite SUVr-cSUVr]) and hippocampus in isolation were used as outcome measures in age- and sex-adjusted models (using LME with random intercept at the center-nested participant’s level and random slope, and linear regression, respectively) given their putative associations with episodic memory [43]. Description of the MRI and PET methods can be found in and elsewhere and in Additional file 2 [26].

## Results

### Characterization of the sample

Table 1 shows the characteristics of the 1097 participants included in the present analyses (65.3% of the MAPT whole sample). Differences at baseline (1-year visit where plasma MCP-1 and Aβ were measured) between MAPT participants included in the present study and those not included are shown in Additional file 3. Mean MCP-1 was 244.96 ± 93.01 (Coefficient of variation: 2.18% ± 1.13%). In total, 274 subjects were classified as MCP-1^+^. These subjects were significantly older, presented higher BMI, worse scores in the CCS, the CDR sum of boxes, and FCSRT immediate free recall (IFR), compared to the MCP-1^−^ group. Median (IQR) follow-up was 3.4 ± 1.0 years.

**Table 1:** Baseline characteristics of the sample

Characteristics	Whole sample Total (*n* = 1097)	Low MCP-1 (*n* = 823)	High MCP-1^a^ (*n* = 274)	Low Aβ42/40 (*n* = 137)	High Aβ42/40^b^ (*n* = 292)
**Women, No. (%)**^**d**^	700 (63.81%)	531 (64.52%)	169 (61.68%)	182 (73.09%)	110 (61.11%)
**Age, years**^**c,d**^	75.30 (4.37)	74.94 (4.21)	76.38 (4.66)	76.49 (4.77)	75.40 (4.44)
**MAPT group allocation, No. (%)**^**d**^					
Omega 3 + MDI group	274 (24.98%)	212 (25.76%)	62 (22.63%)	87 (29.79%)	27 (19.71%)
Omega 3 group	267 (24.34%)	203 (24.67%)	64 (23.36%)	64 (21.92%)	37 (27.01%)
MDI group	277 (25.25%)	198 (24.06%)	79 (28.83%)	77 (26.37%)	27 (19.71%)
Control group	279 (25.43%°	210 (25.52%)	69 25.18%)	64 (21.92%)	46 (33.58%)
**Education, No. (%)**^**d**^					
No diploma	49 (4.54%)	37 (4.57%)	12 (4.43%)	4 (2.96%)	13 (4.51%)
Primary school certificate	179 (16.57%)	133 (16.44%)	46 (16.97%)	38 (28.15%)	47 (16.32%)
Secondary education	354 (32.78%)	256 (31.64%)	98 (36.16%)	52 (38.52%)	84 (29.17%)
High school diploma	168 (15.56%)	135 (16.69%)	33 (12.18%)	10 (7.41%)	51 (17.71%)
University level	330 (30.56%)	248 (30.66%)	82 (30.26%)	31 (22.96%)	93 (32.29%)
**Body mass index**^**c,e**^	26.21 (4.05)	26.03 (3.95)	26.75 (4.32)	26.11 (3.51)	26.58 (4.23)
**Composite cognitive score**^**c,f**^	0.023 (0.69)	0.05 (0.67)	−0.05 (0.75)	−0.22 (0.73)	−0.11 (0.75)
**CDR Sum of boxes, range 0–18**^**c**^	0.39 (0.59)	0.35 (0.55)	0.49 (0.69)	0.53 (0.66)	0.47 (0.67)
**CDR status, No. (%)**^**c**^					
No cognitive impairment, CDR score, 0	580 (52.97%)	456 (55.47%)	124 (45.42%)	151 (98.69%)	86 (98.85%)
Mild cognitive impairment, CDR score, 0.5	509 (46.48%)	362 (44.04%)	147 (53.85%)	2 (1.31%)	1 (1.15%)
Major cognitive impairment, CDR score, ≥ 1	6 (0.55%)	4 (0.49%)	2 (0.73%)	0 (0%)	0 (0%)
**MMSE score, range 0–30**	28.07 (1.81)	28.10 (1.74)	27.98 (2.01)	27.61 (1.88)	27.92 (1.92)
**FCSRT free recall, range 0–48**^**c,d**^	30.38 (7.52)	30.77 (7.17)	29.23 (8.39)	27.54 (8.42)	29.25 (7.86)
**FCSRT total recall, range 0–48**	45.72 (3.80)	45.83 (3.56)	45.40 (4.42)	44.45 (4.85)	45.38 (4.71)
**FCSRT free delayed recall, range 0–16**	11.48 (2.99)	11.53 (2.90)	11.33 (3.26)	10.47 (3.48)	10.95 (3.08)
**FCSRT total delayed recall, range 0–16**	15.52 (1.25)	15.53 (1.22)	15.46 (1.33)	15.19 (1.55)	15.37 (1.58)
**APOE ε4 genotype, No. (%)**^**d**^					
APOE ε4 carriers	226 (22.89%)	168 (20.4%)	58 (21.2%)	45 (33.83%)	58 (22.83%)
Non-APOE ε4 carriers	761 (77.11%)	572 (79.6%)	189 (79.8%)	88 (66.17%)	196 (77.17%)

*Abbreviations*: *Aβ* Amyloid-β, *ADCS-ADL* Alzheimer Disease Cooperative Study–Activities of Daily Living, *APOE* Apolipoprotein E, *CDR* Clinical Dementia Rating, *MDI* Multi-Domain Intervention, *MMSE* Mini-Mental State Examination

^a^High-plasma MCP-1 defined as values in the 4th quartile

^b^Low-plasma Aβ42/40 defined as values ≤ 0.107

^c^*P* < .05 based on Student *t* test or Pearson *χ*^2^ test (between MCP-1 groups)

^d^*P* < .05 based on Student *t* test or Pearson *χ*^2^ test (between Aβ42/40 groups)

^e^Body mass index calculated as weight in kilograms divided by height in meters squared

^f^Based on the *z*-score of 4 cognitive tests (free and total recall of the Free and Cued Selective Reminding test, 10 MMSE orientation items, Digit Symbol Substitution Test, and Category Naming Test)

### Evolution in cognitive outcomes according to MCP-1 plasma levels

As a continuous variable, baseline plasma MCP-1 levels were not prospectively associated to the rate of change of any of the overall cognitive performance outcomes. Regarding domain-specific cognitive functions, MCP-1 was associated with the worsening of episodic memory over 4 years of follow-up, specifically in the FCSRT: delayed free recall (DFR) (*β* = −0.003, 95%CI = −0.005, −0.001; *p* = 0.041) and delayed total recall (DTR) (*β* = −0.001, 95%CI = −0.003, −0.00005 *p* = 0.041). No further associations were found.

In the categorical approach, the MCP-1^+^ group showed a greater cognitive decline according to the CCS at the 4-year follow-up and the CDR sum of boxes score at the 2-year time-point, but this difference did not persist for 3-year and 4-year evolution (Table 2).

**Table 2:** Evolution in overall cognitive outcomes, executive function and attention according to plasma MCP-1 status

	Low-plasma MCP-1^**a**^	High-plasma MCP-1	Between-group difference^**b**^	***P*** value
	Within-group evolution Estimated mean (95% CI)^**c**^	Within-group evolution Estimated mean (95% CI)	Estimated difference (95%CI)	
**Cognitive Composite Score**^**d**^**,*n*= 1079**				
12 months	−0.06 (−0.15, 0.03)	−0.13 (−0.19, −0.08)	−0.08 (−0.17, 0.02)	0.114
24 months	−0.05 (−0.15, 0.04)	−0.18 (−0.18, −0.06)	−0.07 (−0.17, 0.03)	0.196
36 months	−0.14 (−0.24, −0.04)	−0.24 (−0.31, −0.17)	−0.10 (−0.21, 0.02)	0.091
48 months	−**0.18 (**−**0.27,** −**0.08)**	−**0.31 (**−**0.39,** −**0.24)**	−**0.14 (**−**0.26,** −**0.02)**	**0.023**
**MMSE,*n*= 1080**				
12 months	−0.04 (−0.30, −0.22)	−0.15 (−0.37, 0.07)	−0.11 (−0.38, 0.16)	0.418
24 months	−0.03 (−0.29, 0.23)	−0.16 (−0.39, 0.08)	−0.12 (−0.41, 0.16)	0.386
36 months	−0.21 (−0.48, 0.06)	−0.18 (−0.44, 0.08)	0.03 (−0.29, 0.34)	0.853
48 months	−0.15 (−0.42, 0.13)	−0.30 (−0.57, −0.02)	−0.15 (−0.48, 0.18)	0.382
**CDR sum of boxes,*n*= 1080**				
12 months	**0.12 (0.07, 0.16)**	**0.24 (0.15, 0.34)**	**0.13 (0.03, 0.23)**	**0.014**
24 months	**0.13 (0.07, 0.18)**	**0.32 (0.20, 0.43)**	**0.19 (0.06, 0.32)**	**0.004**
36 months	0.23 (0.16, 0.30)	0.36 (0.22, 0.51)	0.14 (−0.03, 0.30)	0.098
48 months	0.34 (0.26, 0.41)	0.52 (0.35, 0.69)	0.18 (−0.01, 0.38)	0.061
**Executive function composite score**^**e**^**,*n*= 1068**				
12 months	−0.03 (−0.07, 0.004)	−0.07 (−0.18, 0.04)	−0.04 (−0.15, 0.08)	0.544
24 months	−0.07 (−0.11, −0.03)	−0.11 (−0.23, 0.004)	−0.05 (−0.17, 0.07)	0.452
36 months	−0.12 (−0.17, −0.07)	−0.18 (−0.30, −0.05)	−0.06 (−0.19, 0.07)	0.381
48 months	−0.15 (−0.19, −0.10)	−0.21 (−0.34, −0.08)	−0.06 (−0.20, 0.08)	0.379
**Attention score**^**f**^**,*n*= 1080**				
12 months	−0.03 (−0.07, 0.004)	−0.07 (−0.18, 0.04)	−0.04 (−0.15, 0.08)	0.544
24 months	−0.07 (−0.11, −0.03)	−0.11 (−0.23, 0.004)	−0.05 (−0.17, 0.07)	0.452
36 months	−0.12 (−0.17, −0.07)	−0.18 (−0.30, −0.05)	−0.06 (−0.19, 0.07)	0.381
48 months	−0.15 (−0.19, −0.10)	−0.21 (−0.34, −0.08)	−0.06 (−0.20, 0.08)	0.379

Significant associations in bold. Models were adjusted by sex, age, BMI, MAPT group, CDR status at baseline, GDS score, and ApoE ε4 genotype

*Abbreviations*: *MCP-1* Monocyte Chemoattractant Protein-1, *MMSE* Mini-Mental State Examination, *CDR* Clinical Dementia Rating

^a^High MCP-1 defined as values in the 4th quartile (> 251 pg/mL)

^b^Negative values for within-group differences mean cognitive decline, except for CDR sum of boxes (for which it is given by positive value)

^c^Negative values for between-group differences indicate more pronounced cognitive decline among the high-plasma MCP-1 group, except for CDR sum of boxes (for which it is given by positive values)

^d^Based on the mean *z*-score of 4 cognitive tests (free and total recall of the Free and Cued Selective Reminding test, 10 MMSE orientation items, Digit Symbol Substitution Test, and Category Naming Test)

^e^Based on the mean *z*-score of 3 executive function tests (Controlled Oral Word Association Test, the Category Naming Test and the Trail Making Test-Part B)

^f^Based on the mean *z*-score of 2 attention tests (Digit Symbol Test and the Trail Making Test-Part A)

As displayed in Table 3, for domain-specific cognitive functions the MCP-1^+^ group showed a greater decline in the episodic memory domain (IFR, DFR, and DTR scores) compared to MCP-1^−^ subjects. Whereas immediate total recall (ITR) declined significantly more in the MCP-1^+^ group over the first 2 years of follow-up (*β* = −0.68, 95%CI = −1.33, −0.02; *p* = 0.042), this association was not found onwards.

**Table 3:** Evolution in memory outcomes according to plasma MCP-1 status

	Low plasma MCP-1^**a**^	High plasma MCP-1	Between-group difference^**b**^	***P*** value
	Within-group evolution Estimated mean (95% CI)^**c**^	Within-group evolution Estimated mean (95% CI)	Estimated difference (95%CI)	
**FCSRT free recall,*n*= 1079**				
12 months	−1.28 (−1.66, −0.91)	−1.93 (−3.00, −0.85)	−0.65 (−1.73, 0.44)	0.243
24 months	−0.70 (−1.11, 0.30)	−1.56 (−2.69, −0.44)	−0.86 (−2.01, 0.29)	0.142
36 months	−1.56 (−2.03, −1.10)	−2.82 (−4.03, −1.60)	−1.25(−2.51, 0.01)	0.051
48 months	−1.82 (−2.33, −1.32)	−3.24 (−4.52, −1.96)	−1.42 (−2.76, −0.06)	**0.039**
**FCSRT total recall,*n*= 1079**				
12 months	−0.76 (−0.98, −0.55)	−1.63 (−2.22, −1.05)	−0.87 (−1.46, −0.27)	**0.004**
24 months	−0.40 (−0.64, −0.16)	−1.07 (−1.71, −0.44)	−0.68 (−1.33, −0.02)	**0.042**
36 months	−1.16 (−1.45,−0.88)	−1.81 (−2.52, −1.11)	−0.65 (−1.40, −0.10)	0.089
48 months	−1.09 (−1.42, −0.77)	−1.59 (−2.37, −0.82)	−0.50 (−1.33, 0.33)	0.240
**FCSRT free delayed recall,*n*= 1079**				
12 months	−0.13 (−0.30, 0.04)	−0.60 (−0.96, −0.24)	−0.47 (−0.84, −0.10)	0.090
24 months	−0.17 (−0.35, 0.01)	−0.52 (−0.90, −0.15)	−0.35 (−0.74, 0.04)	0.206
36 months	−**0.32 (**−**0.52,** −**0.11)**	−**0.82 (**−**1.23,** −**0.41)**	−**0.50 (**−**0.04,** −**0.07)**	**0.045**
48 months	−**0.54 (**−**0.75,** −**0.32)**	−**1.06 (1**−**49,** −**0.63)**	−**0.52 (**−**0.98,** −**0.06)**	**0.024**
**FCSRT total delayed recall,*n*= 1079**				
12 months	−**0.10 (**−**0.18, 0.02)**	−**0.34 (**−**0.53,** −**0.15)**	−**0.24 (**−**0.44,** −**0.05)**	**0.014**
24 months	−**0.10 (**−**0.19,** −**0.02)**	−**0.38 (**−**0.59,** −**0.17)**	−**0.28 (**−**0.50,** −**0.05)**	**0.015**
36 months	−0.32 (−0.42, −0.21)	−0.46 (−0.71, −0.22)	−0.15 (−0.41, 0.12)	0.284
48 months	−**0.35 (**−**0.47,** −**0.23)**	−**0.77 (**−**1.05,** −**0.49)**	−**0.42 (**−**0.72,** −**0.12)**	**0.007**

Significant associations in bold. Models were adjusted by sex, age, BMI, MAPT group, CDR status at baseline, GDS score, and ApoE ε4 genotype

*Abbreviations*: *MCP-1* Monocyte Chemoattractant Protein-1, *FCSRT* Free and Cued Selective Reminding Test

^a^High MCP-1 defined as values in the 4th quartile (> 251 pg/mL)

^b^Negative values for within-group differences mean cognitive decline

^c^Negative values for between-group differences indicate more pronounced cognitive decline among the high plasma MCP-1 group

Removal of ApoE ε4 genotype status from the models slightly modified the results. CCS declines observed in both MCP-1 groups were no longer observed. Concerning memory outcomes in the FCSRT, associations remained significant, but the differences between groups for the IFR, DFR, and DTR scores were observed at 4 years of follow-up (Additional files 4 and 5).

### Evolution in cognitive outcomes according to combined MCP-1 / Aβ_42/40_ plasma levels

**Fig. 1:**
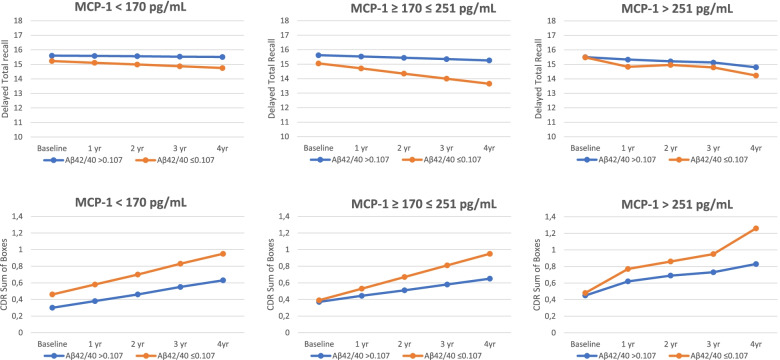
Interactive longitudinal associations of Aβ42/40 and MCP-1 status on CDR Sum of boxes score

The *Aβ*_*42/40*_*× MCP-1 × time* interaction was significant in models with CDR sum of boxes and FCSRT DTR as dependent variables, indicating that greater levels in the MCP-1 might exacerbate the existing association between lower levels of Aβ_42/40_ and the worsening evolution in these two outcomes (CDR sum of boxes: *β* = −0.0005; 95% CI = −0.0009, −0.0002; *p* = 0.041; FCSRT DTR: *β* = 0.0005; 95% CI = 0.0002, 0.0009; *p* = 0.005). Figure 1 graphically displays the evolution in CDR sum of boxes and FCSRT DTR according to Aβ_42/40_ status at different levels of MCP-1.

When combining Aβ_42/40_ and MCP-1 categories, both the Aβ_42/40_^+^/MCP-1^−^ and the Aβ_42/40_^+^/MCP-1^+^ groups experienced significant worsening on the CCS score compared to the Aβ_42/40_^−^/MCP-1^−^ group (between-group difference: *β* = −0.40, 95%CI = −0.59, −0.21; *p* < 0.001 and *β* = −0.41, 95%CI = −0.65, −0.17; *p* < 0.001, respectively), whereas only the Aβ_42/40_^+^/MCP-1^−^ was associated with worsening on the MMSE score (between-group difference: *β* = −0.69, 95%CI = −1.23, −0.15; *p* = 0.012) (Table 4). Both the Aβ_42/40_^+^/MCP-1^−^ and the Aβ_42/40_^+^/MCP-1^+^ groups showed significant progression in CDR sum of boxes score (*β* = 0.37, 95%CI = 0.06, 0.68; *p* = 0.019 and *β* = 0.54, 95%CI = 0.15, 0.93; *p* = 0.007 respectively) (Table 4).

**Table 4:** Evolution in overall cognitive outcomes, executive function and attention according to combined plasma MCP-1 and Aβ42/40 status

Period	Aβ42/40^**−a**^/MCP1^**−b**^ ***n*** = 195	Aβ42/40^**−**^/MCP1^**+**^ ***n*** = 85	Aβ42/40^**+**^ /MCP1^**−**^ ***n*** = 97	Aβ42/40^**+**^ /MCP1^**+**^ ***n*** = 52
	Between-group difference (95% CI)^c^	Between-group difference (95% CI)^c^	Between-group difference (95% CI)^c^	Between-group difference (95% CI)^c^
**Cognitive Composite Score**^**d**^**,*n*= 383**				
12 months	−0.09 (−0.12, −0.05)	−0.08 (−0.18, −0.03)	−0.24 (−0.39, −0.10)***	−0.23 (−0.42, −0.04)**
24 months	−0.06 (−0.10, −0.03)	−0.08 (−0.19, 0.03)	−0.43 (−0.58, −0.27)***	−0.28 (−0.48, −0.07)**
36 months	−0.16 (−0.20, −0.12)	−0.28 (−0.24, −0.00)	−0.39 (−0.56, −0.21)***	−0.23 (−0.44, −0.01)*
48 months	−0.19 (−0.24, −0.15)	−0.13 (−0.26, −0.004)	−0.40 (−0.59, −0.21)***	−0.41 (−0.65, −0.17)***
**MMSE,*n*= 383**				
12 months	−0.12 (−0.26, 0.01)	−0.09 (−0.38, 0.21)	−0.45 (−0.87, −0.04)*	−0.50 (−1.03, 0.02)
24 months	−0.06 (−0.20, 0.08)	−0.21 (−0.52, 0.10)	−0.99 (−1.43, −0.55)***	−0.36 (−0.91, 0.20)
36 months	−0.28 (−0.43, −0.13)	0.01 (−0.33, 0.36)	−0.60 (−1.11, −0.08)*	−0.28 (−0.90, 0.35)
48 months	−0.21 (−0.37,−0.05)	−0.13 (−0.49, 0.23)	−0.69 (−1.23, −0.15)*	−0.66 (−1.35, 0.02)
**CDR sum of boxes,*n*= 383**				
12 months	0.14 (0.08, 0.20)	0.11 (−0.002, 0.22)	0.08 (−0.08, 0.24)	0.24 (0.04, 0.44)*
24 months	0.13 (0.06, 0.20)	0.17 (0.03, 0.31)	0.13 (−0.07, 0.34)	0.34 (0.08, 0.59)*
36 months	0.23 (0.15, 0.31)	0.12 (−0.06, 0.30)	0.22 (−0.05, 0.48)	0.31 (−0.01, 0.64)
48 months	0.33 (0.23, 0.43)	0.15 (−0.07, 0.36)	0.37 (0.06, 0.68)*	0.54 (0.15, 0.93)*
**Executive function composite score**^**e**^**,*n*= 376**				
12 months	−0.02 (−0.05, 0.02)	−0.05 (−0.17, 0.06)	−0.32 (−0.49, −0.15)***	−0.13 (−0.35, 0.08)
24 months	−0.04 (−0.08, −0.004)	−0.07 (−0.17, 0.06)	−0.34 (−0.52, −0.17)***	−0.20 (−0.43, 0.02)
36 months	−0.10 (−0.14, −0.05)	−0.08 (−0.21, 0.06)	−0.39 (−0.58, −0.19)***	−0.24 (−0.48, 0.01)
48 months	−0.12 (−0.17, −0.07)	−0.10 (−0.24, 0.05)	−0.41 (−0.62, −0.20)***	−0.21 (−0.48, 0.06)
**Attention score**^**f**^**,*n*= 383**				
12 months	−0.03 (−0.05, 0.00)	0.00 (−0.09, 0.09)	−0.11 (−0.24, 0.02)	−0.13 (−0.29, 0.03)
24 months	−0.03 (−0.06, −0.01)	−0.01 (−0.11, 0.08)	−0.17 (−0.30, −0.03)	−0.21 (−0.38, −0.04)*
36 months	−0.10 (−0.13, −0.07)	−0.04 (−0.14, 0.06)	−0.12 (−0.27, 0.02)	−0.23 (−0.41, −0.05)*
48 months	−0.13 (−0.17, −0.10)	−0.04 (−0.14, 0.07)	−0.19 (−0.34, −0.04)*	−0.29 (−0.48, −0.09)**

*p value < 0.05; ** *p* value < 0.001; ***p value < 0.001: significant differences in the evolution of the outcomes (Aβ42/40^−^/MCP1^−^ as reference group)

^#^*p* value < 0.05; ^##^*p* value < 0.001; ^###^*p* value < 0.001: significant difference in the evolution of the outcomes between Aβ42/40^+^ /MCP1^−^ and Aβ42/40^+^/MCP1^+^ groups

Models were adjusted by sex, age, BMI, MAPT group, CDR status at baseline, GDS score, and ApoE ε4 genotype

*Abbreviations*: *Aβ42/40* β-amyloid 42aa isoform/β-amyloid 40aa isoform ratio, *MCP-1* Monocyte Chemoattractant Protein-1, *MMSE* Mini-Mental State Examination, *CDR* Clinical Dementia Rating

^a^Abnormal Aβ42/40 defined as values ≥ 107 pg/mL

^b^Abnormal MCP-1 defined as values in the 4th quartile (> 251 pg/mL)

^c^Negative values indicate worsening performance along follow-up, except for CDR sum of boxes (for which it is given by positive values)

^d^Based on the *z*-score of 4 cognitive tests (free and total recall of the Free and Cued Selective Reminding test; 10 MMSE orientation items; Digit Symbol Substitution Test, and Category Naming Test)

^e^Based on the *z*-score of 3 executive function tests (Controlled Oral Word Association Test, the Category Naming Test and the Trail Making Test-Part B)

^f^Based on the *z*-score of 2 attention tests (Digit Symbol Test and the Trail Making Test-Part A)

Significant greater associations between Aβ_42/40_/MCP-1 status and executive function composite score were found only in the Aβ_42/40_^+^/MCP-1^−^ group (*β* = −0.41, 95%CI = −0.62, −0.20; *p* < 0.001), whereas for the attention composite score, both Aβ_42/40_^+^/MCP-1^−^ and Aβ_42/40_^+^/MCP-1^+^ groups showed a greater decline compared to the reference group (Table 4). With regard to the memory outcomes, the Aβ_42/40_^+^/MCP-1^−^ group showed significant greater worsening in the performance across the four outcomes (between-group difference IFR: *β* = −4.22, 95%CI = −7.71, −2.17; *p* < 0.001, ITR: *β* = −3.14, 95%CI = −4.48, −1.81; *p* < 0.001, DFR: *β* = −1.90, 95%CI = −2.74, −1.06; *p* < 0.001 and DTR: *β* = −1.20, 95%CI = −1.69, −0.72; *p* < 0.001) compared to the Aβ_42/40_^−^/MCP-1^−^ group over the 4 years of follow-up. Similarly, the Aβ_42/40_^+^/MCP-1^+^ experienced greater declines in the four FCSRT outcomes compared to the reference group (Table 5).

**Table 5:** Evolution in memory outcomes according to combined plasma MCP-1 and Aβ42/40 status

Period	Aβ42/40^**−a**^/MCP1^**−b**^ ***n*** = 195	Aβ42/40^**−**^/MCP1^**+**^ ***n*** = 85	Aβ42/40^**+**^ /MCP1^**−**^ ***n*** = 97	Aβ42/40^**+**^ /MCP1^**+**^ ***n*** = 52
	Between-group difference (95% CI)^c^	Between-group difference (95% CI)^c^	Between-group difference (95% CI)^c^	Between-group difference (95% CI)^c^
**FCSRT free recall,*n*= 383**				
12 months	−1.10 (−1.47, −0.72)	−0.61 (−1.75, 0.52)	−3.26 (−4.87, −1.65) *	−2.74 (−4.76, −0.73)**
24 months	−0.46 (−0.86, −0.06)	−0.95 (−2.14, 0.25)	−4.24 (−5.94, −2.54) ***	−2.36 (−4.51, −0.21)*
36 months	−1.30 (−1.76, −0.84)	−1.58 (−2.90, −0.27)*	−4.46 (−7.64, −2.55) ***	−1.81 (−4.17, 0.54)
48 months	−1.47 (−1.97, −0.98)	−1.51 (−2.90, −0.11)*	−4.22 (−7.71, −2.17) ***	−3.31 (−5.89, −0.73)*
**FCSRT total recall,*n*= 383**				
12 months	−0.60 (−0.84, −0.36)	−0.95 (−1.61, −0.30)	−2.24 (−3.17, −1.30)***	−1.88 (−3.06, −0.71)***
24 months	−0.18 (−0.45, 0.08)	−0.71 (−1.43, 0.004)	−2.29 (−3.31, −1.26)***	−1.92 (−3.22, −0.62)**
36 months	−0.92 (−1.24, −0.61)	−0.63 (−1.45, 0.18)	−2.32 (−3.53, −1.12)***	−2.08 (−3.56, −0.60)**
48 months	−0.81 (−1.17, −0.46)	−0.45 (−1.35, 0.45)	−3.14 (−4.48, −1.81)***	−2.54 (−4.22, −0.86)**
**FCSRT free delayed recall,*n*= 383**				
12 months	−0.10 (−0.27, 0.06)	−0.25 (−0.70, 0.21)	−1.12 (−1.76, −0.48)***	−1.48 (−2.29, −0.68)**
24 months	−0.11 (−0.28, 0.07)	−0.28 (−0.76, 0.20)	−1.38 (−2.06, −0.70)***	−1.14 (−2.01, −0.28)*
36 months	−0.24 (−0.44, −0.04)	−0.61 (−1.14, −0.07)*	−1.37 (−2.15, −0.59)***	−0.86 (−1.82, 0.08)
48 months	−0.38 (−0.60, −0.17)	−0.69 (−1.26, −0.13)*	−1.90 (−2.74, −1.06)***	−1.28 (−2.33, −0.23)*
**FCSRT total delayed recall,*n*= 383**				
12 months	−0.07 (−0.15, 0.01)	−0.22 ( −0.43, −0.01)*	−0.57 (−0.87, −0.26)***	−0.66 (−1.04, −0.28)***
24 months	−0.06 (−0.15, 0.03)	−0.32 (−0.57, −0.08)**	−0.72 (−1.07, −0.37)***	−0.51 (−0.95, −0.07)*
36 months	−0.25 (−0.36, −0.14)	−0.20 (−0.49, −0.08)	−1.00 (−1.43, −0.58)***	−0.46 (−0.98, 0.07)
48 months	−0.26 (−0.39 , −0.13)	−0.45 (−0.78, −0.13)**	−1.20 (−1.69, −0.72)***	−0.93 (−1.54, −0.31)**

*p value < 0.05; **p value < 0.001; ***p value < 0.001: significant differences in the evolution of the outcomes (Aβ42/40^-^/MCP1^−^ as reference group)

^#^*p* value < 0.05; ^##^*p* value < 0.001;^###^*p* value < 0.001: significant difference in the evolution of the outcomes between Aβ42/40^+^ /MCP1^−^ and Aβ42/40^+^ /MCP1^+^ groups

Models were adjusted by sex, age, BMI, MAPT group, CDR status at baseline, GDS score, and ApoE ε4 genotype

*Abbreviations*: *Aβ42/40* β-amyloid 42aa isoform/β-amyloid 40aa isoform ratio, *FCSRT* Free and Cued Selective Reminding Test, *MCP-1* Monocyte Chemoattractant Protein-1

^a^Abnormal Aβ42/40 defined as values ≥ 107 pg/mL

^b^Abnormal MCP-1 defined as values in the 4th quartile (> 251 pg/mL)

^c^Negative values indicate worsening performance along follow-up

We did not find significant differences for any FCSRT outcome between Aβ_42/40_^+^/MCP-1^−^ and Aβ_42/40_^+^/MCP-1^+^ (Table 5). Additional files 5 and 6 show the within-group evolution in the different outcomes. Sensitivity analyses removing ApoE ε4 genotype status from the models yielded similar results (Additional files 7 and 8).

### Post hoc analyses

Based on the findings suggesting a domain-specific association between MCP-1 and episodic memory, we undertook exploratory analyses to test whether baseline plasma MCP-1 levels were associated with hippocampus volume changes and cSUVr and hippocampal β-amyloid load. MCP-1 was significantly associated with hippocampal atrophy (*n* = 299) when used as continuous (*β* = −0.0000005, 95%CI = −0.0000009, −0.00000007; *p* = 0.022), whereas in the categorical approach, the MCP-1^+^ group did not show a greater hippocampal atrophy over the follow-up (mean = 966.1 ± 111 days), compared to MCP-1^−^ (between-group difference: *β* = −0.04, 95%CI = −0.10, 0.01; *p* = 0.107).

With regard to cross-sectional PET-scan β-amyloid load (*n* = 193), continuous plasma MCP-1 was neither cross-sectionally associated with hippocampal (*β* = −0.00008, 95%CI = −0.0002, 0.00008; *p* = 0.299) or cSUVr (*β* = −0.0001, 95%CI = −0.0004, 0.0001; *p* = 0.337), whereas in the categorical approach, MCP-1^+^ was associated with lower hippocampal SUVr (*β* = −0.04, 95%CI = −0.07, −0.002; *p* = 0.036), but not with cSUVr (*β* = −0.00002, 95%CI = −0.0001, 0.0001; *p* = 0.797).

## Discussion

Our results showed that higher levels of MCP-1 were associated with greater overtime declines on both overall and domain-specific cognitive functions. Domain-specific analysis revealed that higher plasma MCP-1 levels were consistently associated with more pronounced decreases in the episodic memory performance; exploratory analysis found MCP-1 was notably associated with overtime hippocampal atrophy, suggesting MCP-1 association with cognition might be mediated by changes in the hippocampus structure. The association between MCP-1 and cognitive decline was strongest in those with amyloid plaques, as measured by blood plasma Aβ42/40.

Our findings linking higher plasma MCP-1 and cognitive decline are compatible with available research describing its pathophysiological involvement in neurodegeneration as well as with results of recent animal- and human-based studies addressing its association with cognitive decline [44]. It is possible that peripheral immunologic alterations mirror CNS neuroinflammatory processes [45, 46], what would allow to monitor cognitive decline-related pathophysiological changes occurring before overt clinical manifestations [47]. In fact, it has been proposed that peripheral-mediated processes might not only constitute a reflection of CNS inflammation, but also exacerbate CNS glial cell activation. This occurs in the presence of an age- and inflammation-related increase in blood-brain barrier permeability, allowing the leakage of circulating inflammatory factors and immune cells into CSF and brain parenchyma [48]. In this sense, MCP-1, given its potential role as a regulator of the migration and infiltration into the CNS, might have a prominent utility as a neuroinflammatory marker [16], even when measured in plasma.

Previous murine-based studies have suggested that aging is characterized by increased circulating MCP-1 and that elevated MCP-1 is associated with age-related decline in neurogenesis and subsequent worse performance in memory domains [17, 18]. Contrary to our findings, the first human study exploring longitudinal associations of MCP-1 with Alzheimer’s disease (AD) progression showed that CSF but not plasma MCP-1 was elevated in prodromal AD compared to non-prodromal AD individuals with mild-cognitive impairment (MCI), and that CSF MCP-1 was associated to greater cognitive decline in subjects developing AD [34]. Notwithstanding, Lee et al. showed increasing levels of plasma MCP-1 along the AD continuum (from healthy controls to severe AD dementia—CDR = 3) in an outpatient sample of older adults. They further showed that, among MCI and AD participants, MCP-1 levels were associated with 2-year declines in cognitive function evaluated through the MMSE [24]. In line with our findings, Bettcher et al. found associations of plasma MCP-1 levels with episodic memory cross-sectionally in cognitively impaired subjects [20] and longitudinally [23] in asymptomatic older adults; no associations were observed with other cognitive functions. Our findings, alongside Bettcher et al.’s results, suggest the MCP-1-cognitive function associations may be specific to episodic memory function in older adults. The results of our exploratory, post hoc neuroimaging analyses are compatible with previous research linking neuroinflammatory processes and hippocampal volumes in different samples of older adults [49]; since changes in hippocampal volume are linked to episodic memory performance [43], it is possible that the MCP-1-episodic memory associations might be mediated by hippocampal atrophy.

Surprisingly, the magnitude of the associations with cognitive decline was higher in the Aβ42/40+/MCP-1− compared to the Aβ42/40+/MCP-1+. Although statistically non-significant, this unexpected association deserves further investigations since it raises questions about the role of MCP-1 on cognitive function in the context of β-amyloid deposition, especially on episodic memory. To the best of our knowledge, the sole study investigating the combined interaction of MCP-1 and β-amyloid [19] showed that, in the presence of abnormal CSF tau protein and Aβ_42_/P-tau ratio, increased levels of CSF MCP-1 exacerbated cognitive decline among MCI older adults. Indeed, while we found a modest worsening of both the CDR sum of boxes score and a measure of episodic memory when MCP-1 and Aβ_42/40_ levels were used as continuous, our study failed in finding the added value of MCP-1 when combined with Aβ_42/40_ in the categorical approach. Recent research has revealed that, distinctly to in pre-clinical stages, in MCI and AD, microglial function is impaired [50, 51], exacerbating Aβ deposition [52, 53], tau pathology [54, 55], and synaptic/neuronal loss [53, 56].

In any case, the conflicting findings about the potential interactions between MCP-1 and β-amyloid deposition to determine cognitive declines ask for further research in this topic. Specifically, the exploration of the role of MCP-1 in determining cognitive declines at different stages of cognitive decline/dementia progression should be subject of further research [57]. Importantly, our study expands findings of available studies to a sample of older adults in early stages of cognitive decline for the first time.

Other mechanisms besides β-amyloid deposition are involved in neuroinflammation-mediated cognitive impairment. Indeed, mechanistic research has shown the potential of MCP-1 to promote tau-phosphorylation and neurofibrillary tangle formation [54, 58], a core feature of AD that has been lately shown to be to be more closely related to cognitive evolution than β-amyloid dyshomeostasis in the brain [59]. Potentially, tau-phosphorylation and the formation of intracellular neurofibrillary tangles might partially explain associations between MCP-1 and cognitive outcomes in older adults. Unfortunately, we did not have available data on tau-related markers. This topic needs further investigations.

### Strengths and limitations

Our study presents strengths: its longitudinal nature with a relatively long follow-up and several time-points of data collection, which allow us to know the trajectories of the different cognitive outcome measures; its large sample size compared to previous investigations; and the assessment of both overall and domain-specific cognitive functions.

Nevertheless, our findings should be interpreted considering several limitations. We included a group of relatively healthy and highly educated older adults who participated in a randomized clinical trial; therefore, results may differ in observational studies with demographically diverse populations and generalization of results should be cautious. However, the interventions of the MAPT study did not have significant effects on cognitive function [27]; moreover, all our analyses were adjusted to MAPT group allocation, minimizing potential bias. Although the sample size was substantially reduced in the analyses exploring the combination of MCP-1 and Aβ_42/40_, which might have led to a reduction in statistical power, we still had 429 subjects with available data in both markers, a larger sample than the population of previous investigation [19]. Although the ability of peripheral MCP-1 to reflect its CNS levels is not fully established since this chemokine may be expressed by different tissues, there is some evidence showing MCP-1 levels in plasma and CSF are strongly correlated [46].

## Conclusion

The current study showed that higher plasma MCP-1 levels were associated with declines in both overall and episodic memory cognitive performances in older adults over a 4-year follow-up. Whether MCP-1 levels in the context of low-plasma Aβ_42/40_ ratio may confer an increased risk for cognitive decline remains an open question. Further research is needed to examine the potential associations between longitudinal changes in plasma MCP-1 and cognitive evolution in population-based studies to clarify the validity of this chemokine as a marker of cognitive decline at different disease stages. In addition, the clarification of the directionality and the mechanisms underlying the association between neuroinflammation and cognitive decline at different stages of the disease should be the subject of future research.

## Supplementary Information

**Additional file 1.** Description of features of ELLA System for plasma MCP-1 determination.

**Additional file 2.** Neuroimaging procedures. Description of neuroimaging techniques used in the present study.

**Additional file 3.** Differences between included and excluded participants. Differences in baseline characteristics between MAPT study participants included and excluded in the present study.

**Additional file 4.** Evolution in overall cognitive outcomes, executive function and attention according to plasma MCP-1 status (excluding ApoE ε4 genotype). Mixed-effect linear regression analysis for variation in overall cognitive outcomes, executive function and attention over time according to plasma MCP-1 status among community-dwelling older adults (excluding ApoE ε4 genotype).

**Additional file 5.** Evolution in overall memory outcomes according to plasma MCP-1 status (excluding ApoE ε4 genotype). Mixed-effect linear regression analysis for variation in memory outcomes over time according to plasma MCP-1 status among community-dwelling older adults (excluding ApoE ε4 genotype).

**Additional file 6.** Within group evolution in overall cognitive outcomes, executive function and attention according to plasma MCP-1 status (excluding ApoE ε4 genotype). Within group evolution in memory outcomes over time according to plasma MCP-1 status among community-dwelling older adults (excluding ApoE ε4 genotype).

**Additional file 7.** Within group evolution in memory outcomes, executive function and attention according to plasma MCP-1 status (excluding ApoE ε4 genotype). Mixed-effect linear regression analysis for variation in memory outcomes over time according to plasma MCP-1 status among community-dwelling older adults (excluding ApoE ε4 genotype).

**Additional file 8.** Within group evolution in memory outcomes, executive function and attention according to plasma MCP-1 status (excluding ApoE ε4 genotype). Mixed-effect linear regression analysis for variation in overall cognitive outcomes, executive function and attention over time according to combined plasma Aβ_42/40_^+^ and MCP-1 status among community-dwelling older adults (excluding ApoE ε4 genotype).

**Additional file 9.** Within group evolution in memory outcomes, executive function and attention according to plasma MCP-1 status (excluding ApoE ε4 genotype). Mixed-effect linear regression analysis for variation in memory outcomes over time according to combined plasma Aβ_42/40_^+^ and MCP-1 status among community-dwelling older adults (excluding ApoE ε4 genotype).

## Data Availability

The data that support the findings of this study are available from MAPT Study Group but restrictions apply to the availability of these data, which were used under license for the current study, and so are not publicly available. Data are however available from the authors upon reasonable request and with permission of Nicola Coley (nicola.coley@inserm.fr).
