# Supplementary material for: Plasma MCP-1 and changes on cognitive function in community-dwelling older adults
Source: Alzheimers Res Ther. 2022 Jan 7;14:5. doi: 10.1186/s13195-021-00940-2 (PMC8742409; doi:10.1186/s13195-021-00940-2)
Supplement: Supplementary file 1 — Additional file 1. Description of features of ELLA System for plasma MCP-1 determination. [file 13195_2021_940_MOESM1_ESM.docx]

**Additional file 1. Description of features of ELLA System for plasma MCP-1 and anti-Aβ middomain antibody (HJ5.1, anti-Aβ13-28) conjugated to M-270 Epoxy Dynabeads (Invitrogen)for plasma Aβ42 and Aβ40 determination**

**ELLA System-MCP-1**

Simple Plex™ is an integrated immunoassay system that consists of a disposable microfluidic cartridge and an automated analyzer, the Ella instrument. Each sample is analyzed in a unique parallel channel within the cartridge.

Upper Limit of Detection: 5780 pg/mL

Lower Limit of Detection: 1,52 pg/mL

Linearity: 1,02

Coefficient of variation: 6,3%

**anti-Aβ middomain antibody (HJ5.1, anti-Aβ13-28)- Aβ40**

Upper Limit of Detection:1789 pg/ml

Lower Limit of Detection: <7,60 pg/ml

Linearity: 0,998

Coefficient of variation: 6,81%

**anti-Aβ middomain antibody (HJ5.1, anti-Aβ13-28)- Aβ42**

Upper Limit of Detection: 1465 pg/mL

Lower Limit of Detection: < 3.60 pg/mL

Linearity: 0,998

Coefficient of variation: 5,73%
