# Supplementary material for: Plasma MCP-1 and changes on cognitive function in community-dwelling older adults
Source: Alzheimers Res Ther. 2022 Jan 7;14:5. doi: 10.1186/s13195-021-00940-2 (PMC8742409; doi:10.1186/s13195-021-00940-2)
Supplement: Supplementary file 2 — Additional file 2. Neuroimaging procedures. Description of neuroimaging techniques used in the present study. [file 13195_2021_940_MOESM2_ESM.docx]

**Additional File 2.** Methods of neuroimaging

**MRI acquisition and analyses**

The acquisition protocol for brain MRI has been detailed elsewhere (Maltais et al., 2019; Vellas et al., 2014). In brief, the MRI scan was performed within the first 12 months of MAPT study enrollment and two years later, using a standardized protocol in each of the centers (Toulouse, Bordeaux, Montpellier, Limoges, Dijon, Lyon, Foix, Tarbes and Nice). In this study, we included MRI measurement for the total intracranial volume (cm3) and the hippocampus volume (cm3). Quality of each imaging data was assessed, and measures were excluded from our analysis if presenting poor or unreliable quality. The 3D T1-weighted sequence, derived by the SPM5 toolbox (fil.ion.ucl.ac.uk/spm), was used to compute the volumes.

**18-florbetapir PET acquisition and analysis**

PET scans were realized as close as possible to a clinical visit during the 3 years of follow-up of each patient, as previously described (Vellas et al., 2014)(Del Campo et al., 2016). Participants were examined using 5 different hybrid PET-CT scanners, including gone PET CT 690 (GE Healthcare), one Discovery RX VCT (GeneralElectric), 2 True Point HiRez (Siemens Medical Solutions), and one Biograph 4 Emission Duo LSO (Siemens Medical Solutions). All tomographs operated in 3D detection mode. All PET sinograms were reconstructed with an iterative algorithm, with corrections for randomness, scatter, photon attenuation, and decay, which pro-duced images with an isotropic voxel of 2 x 2 x2mm3 and a spatial resolution of approximately 5-mm full width at a half maximum at the field of view center. The acquisition data were processed using the standard package delivered with each acquisition system. All cerebral emission scans began 50 minutes after a mean injection of 4MBq/kg weight of F18-florbetapir. For each subject, 10- or 15-minuteframes were acquired to ensure movement-free image acquisition. A semiautomated quantitative analysis (cortical to cerebellar regional mean standardized uptake value ratio [SUVr]) was applied using the mean signal of 6 predefined anatomically relevant cortical regions of interest (frontal, temporal, parietal, precuneus, anterior cingulate, and posterior cingulate) with the whole cerebellum used as the reference region as previously described (Clark et al., 2011;Fleisher et al., 2011). In this procedure, the F18-florbetapir PET images were coregistered to the F18-florbetapir template provided by AVID company. Quality control based on the semiquantification process was also provided by AVID Lab. To acknowledge the fact that date of realization of PET scans did not matched, we included the difference between PET scan date and the baseline visit in the present study (12 month-follow up in MAPT) as a covariate in the linear regression models.
