# Supplementary material for: Plasma MCP-1 and changes on cognitive function in community-dwelling older adults
Source: Alzheimers Res Ther. 2022 Jan 7;14:5. doi: 10.1186/s13195-021-00940-2 (PMC8742409; doi:10.1186/s13195-021-00940-2)
Supplement: Supplementary file 3 — Additional file 3. Differences between included and excluded participants. Differences in baseline characteristics between MAPT study participants included and excluded in the present study. [file 13195_2021_940_MOESM3_ESM.docx]

**Additional File 3. Baseline differences in characteristics between included and non-included subjects in the present analyses.**

| **Characterstics** | Whole MAPT Sample  (n=1680) | Included Subjects  (n=1097) | Non-included Subjects  (n=583) |
| --- | --- | --- | --- |
| **Women, No. (%)** |  |  |  |
| **Age, y** | 75.33 (4.22) | 75.30 (4.37) | 75.40 (4.53) |
| **Education, No. (%)** |  | | |
| No diploma | 85 (5.17) | 49 (4.54) | 36 (6.39) |
| Primary school certificate | 286 (17.41) | 179 (16.57) | 107 (19.01) |
| Secondary education | 553 (33.66) | 354 (32.78) | 199 (35.35) |
| High school diploma | 242 (14.73) | 168 (15.56) | 74 (13.14) |
| University level | 477 (29.03) | 330 (30.56) | 147 (26.11) |
| **Body Mass Index^c^** | 26.27 (4.04) | 26.21 (4.06) | 26.49 (3.98) |
| **CDR Sum of boxes, range 0-18** | 0.39 (0.58) | 0.39 (0.58) | 0.41 (0.55) |
| **CDR status, No. (%)^b^** |  |  |  |
| No cognitive impairment,  CDR score, 0 | 716 (51.11) | 580 (52.97) | 136 (44.44) |
| Mild cognitive impairment,  CDR score, 0.5 | 677 (48.32) | 509 (46.48) | 168 (54.90) |
| Major cognitive impairment,  CDR score, ≥1 | 8 (0.57) | 6 (0.55) | 2 (0.65) |
| **MMSE score, range 0-30** | 28.03 (1.88) | 28.07 (1.81) | 27.90 (2.12) |
| **FCSRT Free Recall, range 0-48** | 30.38 (7.52) | 30.19 (7.56) | 29.52 (7.68) |
| **FCSRT Total Recall, range 0-48** | 45.69 (3.83) | 45.56 (3.96) | 45.72 (3.80) |
| **FCSRT Free Delayed Recall, range 0-16** | 11.42 (3.02) | 11.48 (2.99) | 11.20 (3.12) |
| **FCSRT Total Delayed Recall, range 0-16** | 15.50 (1.25) | 15.45 (1.36) | 15.52 (1.25) |
| **APOE ε4 genotype, No. (%)** |  |  |  |
| APOE ε4 carriers | 73 (23.32) | 226 (22.90) | 299 (23.00) |
| Non-APOE ε4 carriers | 240 (76.68) | 761 (77.10) | 1001 (77.00) |

Abbreviations: APOE, apolipoprotein E; CDR, Clinical Dementia Rating; FCSRT: Free and Cued Selective Reminding Test; MAPT: Multidomain Alzheimer Preventive Trial; MMSE, Mini-Mental State Examination.

a. High plasma MCP-1 defined as values in the 4^th^ quartile.

b. P < .05 based on T-test or Pearson χ2 test.

c. Body mass index calculated as weight in kilograms divided by height in meters squared.
