# Supplementary material for: Plasma MCP-1 and changes on cognitive function in community-dwelling older adults
Source: Alzheimers Res Ther. 2022 Jan 7;14:5. doi: 10.1186/s13195-021-00940-2 (PMC8742409; doi:10.1186/s13195-021-00940-2)
Supplement: Supplementary file 4 — Additional file 4. Evolution in overall cognitive outcomes, executive function and attention according to plasma MCP-1 status (excluding ApoE ε4 genotype). Mixed-effect linear regression analysis for variation in overall cognitive outcomes, executive function and attention over time according to plasma MCP-1 status among community-dwelling older adults (excluding ApoE ε4 genotype). [file 13195_2021_940_MOESM4_ESM.docx]

**Additional File 4. Mixed-effect linear regression analysis for variation in overall cognitive outcomes, executive function and attention over time according to plasma MCP-1 status among community-dwelling older adults (excluding ApoE ε4 genotype)**

|  | **Low plasma MCP-1^a^** | **High plasma MCP-1** | **Between-group Difference^b^** |  |
| --- | --- | --- | --- | --- |
|  | **Within-group evolution**  **Estimated mean**  **(95% CI)^c^** | **Within-group evolution**  **Estimated mean**  **(95% CI)** | **Estimated difference**  **(95%CI)** | **p-value** |
| **Cognitive Composite Score^d^, n=975** | | | | |
| 12 months | -0.08 (-0.17, 0.01) | -0.13 (-0.18, -0.08) | -0.05 (-0.14, 0.04) | 0.283 |
| 24 months | -0.08 (-0.17, 0.01) | -0.12 (-0.17, -0.06) | -0.04 (-0.14, 0.06) | 0.437 |
| 36 months | -0.16 (-0.26, -0.07) | -0.24 (-0.30, -0.17) | -0.07 (-0.18, 0.04) | 0.204 |
| **48 months** | -0.20 (-0.30, -0.11) | -0.31 (-0.39, -0.24) | -0.11 (-0.23, 0.01) | 0.064 |
| **MMSE, n=975** | | | | |
| 12 months | -0.15 (-0.40, 0.10) | -0.17 (-0.39, 0.04) | -0.02 (-0.28, 0.24) | 0.878 |
| 24 months | -0.18 (-0.43, 0.07) | -0.18 (-0.40, -0.04) | -0.001 (-0.28, 0.27) | 0.955 |
| 36 months | -0.33 (-0.59, -0.06) | -0.19 (-0.44, 0.07) | 0.15 (-0.16, 0.45) | 0.356 |
| **48 months** | -0.28 (-0.55, -0.01) | -0.33 (-0.60, -0.06) | -0.05 (-0.38, 0.28) | 0.770 |
| **CDR sum of boxes, n=975** | | | | |
| **12 months** | 0.10 (0.06, 0.15) | 0.20 (0.11, 0.30) | 0.10 (0.002, 0.20) | **0.045** |
| **24 months** | 0.13 (0.07, 0.18) | 0.28 (0.16, 0.40) | 0.15 (0.03, 0.28) | **0.015** |
| 36 months | 0.22 (0.15, 0.29) | 0.34 (0.19, 0.48) | 0.11 (-0.05, 0.27) | 0.165 |
| 48 months | 0.33 (0.25, 0.42) | 0.48 (0.31, 0.65) | 0.14 (-0.04, 0.33) | 0.132 |
| **Executive function composite score^e^, n=871** | | | | |
| 12 months | -0.03 (-0.06, 0.00) | -0.04 (-0.14, 0.07) | -0.01 (-0.11, 0.09) | 0.908 |
| 24 months | -0.06 (-0.10, -0.03) | -0.09 (-0.20, 0.02) | -0.02 (-0.13, -0.09) | 0.658 |
| 36 months | -0.12 (-0.16, -0.08) | -0.17 (-0.28, -0.05) | -0.04 (-0.16, 0.07) | 0.463 |
| 48 months | -0.15 (-0.20, -0.11) | -0.20 (-0.32, -0.08) | -0.04 (-0.17, 0.08) | 0.507 |
| **Attention score^f^, n=881** | | | | |
| 12 months | -0.03 (-0.07, 0.004) | -0.07 (-0.18, 0.04) | -0.04 (-0.15, 0.08) | 0.544 |
| 24 months | -0.07 (-0.11, -0.03) | -0.11 (-0.23, 0.004) | -0.05 (-0.17, 0.07) | 0.452 |
| 36 months | -0.12 (-0.17, -0.07) | -0.18 (-0.30, -0.05) | -0.06 (-0.19, 0.07) | 0.381 |
| 48 months | -0.15 (-0.19, -0.10) | -0.21 (-0.34, -0.08) | -0.06 (-0.20, 0.08) | 0.379 |

Significant associations in bold. Models were adjusted by sex, age, BMI, MAPT group, CDR status at baseline and GDS score

Abbreviations: MCP-1: Monocyte Chemoattractant Protein-1; MMSE, Mini-Mental State Examination; CDR, Clinical Dementia Rating; MMSE, Mini-Mental State Examination.

a. High MCP-1 defined as values in the 4th quartile (> 251pg/mL).

b. Negative values for within-group differences mean cognitive decline, except for CDR sum of boxes (for which it is given by positive values).

c. Negative values for between-group differences indicate more pronounced cognitive decline among the high plasma MCP-1 group, except for CDR sum of boxes (for which it is given by positive values).

d. Based on the mean Z-score of 4 cognitive tests (free and total recall of the Free and Cued Selective Reminding test; 10 MMSE orientation items; Digit Symbol Substitution Test; and Category Naming Test) .

e. Based on the mean Z-score of 3 executive function tests (Controlled Oral Word Association Test, the Category Naming Test and the Trail Making Test-Part B) e Based on the mean Z-score of 2 attention tests (Digit-Symbol Test and the Trail Making Test-Part A)
