# Supplementary material for: Plasma MCP-1 and changes on cognitive function in community-dwelling older adults
Source: Alzheimers Res Ther. 2022 Jan 7;14:5. doi: 10.1186/s13195-021-00940-2 (PMC8742409; doi:10.1186/s13195-021-00940-2)
Supplement: Supplementary file 5 — Additional file 5. Evolution in overall memory outcomes according to plasma MCP-1 status (excluding ApoE ε4 genotype). Mixed-effect linear regression analysis for variation in memory outcomes over time according to plasma MCP-1 status among community-dwelling older adults (excluding ApoE ε4 genotype). [file 13195_2021_940_MOESM5_ESM.docx]

**Additional File 5. Mixed-effect linear regression analysis for variation in memory outcomes over time according to plasma MCP-1 status among community-dwelling older adults (excluding ApoE ε4 genotype).**

|  | **Low plasma MCP-1^a^** | **High plasma MCP-1** | **Between-group Difference^b^** |  |
| --- | --- | --- | --- | --- |
|  | **Within-group evolution**  **Estimated mean**  **(95% CI)^c^** | **Within-group evolution**  **Estimated mean**  **(95% CI)** | **Estimated difference**  **(95%CI)** | **p-value** |
| **FCSRT Free Recall, n=881** | | | | |
| 12 months | -1.19 (-1.53, -0.84) | -1.76 (-2.75, -0.77) | -0.57 (-1.57, 0.44) | 0.268 |
| 24 months | -0.71 (-1.07, -0.34) | -1.31 (-2.34, -0.27) | -0.60 (-1.66, 0.45) | 0.262 |
| 36 months | -1.50 (-1.93, -1.08) | -2.66 (-3.77, -1.55) | -1.16 (-2.31, 0.001) | 0.054 |
| **48 months** | -1.70 (-2.16, -1.24) | -3.00 (-4.18, -1.82) | 1.30 (-2.54, -0.06) | **0.039** |
| **FCSRT Total Recall, n=881** | | | | |
| 12 months | -0.80 (-1.01, -0.59) | -1.36 (-1.94, -0.78) | -0.56 (-1.15, 0.03) | 0.062 |
| 24 months | -0.43 (-0.66, -0.20) | -0.86 (-1.48, -0.24) | -0.43 (-1.07, 0.22) | 0.192 |
| 36 months | -1.19 (-1.46, -0.91) | -1.60 (-2.29, -0.90) | -0.41 (-1.15, 0.33) | 0.275 |
| **48 months** | -1.17 (-1.48, -0.85) | -1.36 (-2.12, -0.59) | -0.20 (-1.01, 0.63) | 0.646 |
| **FCSRT Free Delayed Recall, n=881** | | | | |
| **12 months** | -0.13 (-0.28, 0.02) | -0.49 (-0.90, -0.08) | -0.26 (-0.66, 0.14) | 0.213 |
| **24 months** | -0.16 (-0.33, 0.001) | -0.44 (-0.87, -0.02) | -0.20 (-0.63, 0.22) | 0.349 |
| 36 months | -0.29 (-0.48, -0.10) | -0.79 (-1.25, -0.32) | -0.45 (-0.92, 0.02) | 0.062 |
| 48 months | -0.48 (-0.69, -0.28) | -1.08 (-1.57, -0.59) | -0.57 (-1.07, -0.06) | **0.028** |
| **FCSRT Total Delayed Recall, n=881** | | | | |
| 12 months | -0.12 (-0.19, -0.05) | -0.23 (-0.42, -0.05) | -0.12 (-0.31, 0.08) | 0.246 |
| 24 months | -0.12 (-0.20, -0.03) | -0.28 (-0.49, -0.07) | -0.16 (-0.38, 0.06) | 0.150 |
| 36 months | -0.31 (-0.41, -0.21) | -0.35 (-0.59, -0.11) | -0.04 (-0.30, 0.22) | 0.751 |
| 48 months | -0.36 (-0.47, -0.24) | -0.70 (-0.97, -0.42) | -0.34 (-0.63, -0.04) | **0.025** |

Significant associations in bold. Models were adjusted by sex, age, BMI, MAPT group, CDR status at baseline, GDS score and ApoE ε4 genotype

Abbreviations: MCP-1: Monocyte Chemoattractant Protein-1. FCSRT, Free and Cued Selective Reminding Test.

a. High MCP-1 defined as values in the 4th quartile (>251 pg/mL).

b. Negative values for within-group differences mean cognitive decline.

c. Negative values for between-group differences indicate more pronounced cognitive decline among the high plasma MCP-1 group.
