# Supplementary material for: Plasma MCP-1 and changes on cognitive function in community-dwelling older adults
Source: Alzheimers Res Ther. 2022 Jan 7;14:5. doi: 10.1186/s13195-021-00940-2 (PMC8742409; doi:10.1186/s13195-021-00940-2)
Supplement: Supplementary file 6 — Additional file 6. Within group evolution in overall cognitive outcomes, executive function and attention according to plasma MCP-1 status (excluding ApoE ε4 genotype). Within group evolution in memory outcomes over time according to plasma MCP-1 status among community-dwelling older adults (excluding ApoE ε4 genotype). [file 13195_2021_940_MOESM6_ESM.docx]

**Additional File 6. Mixed-Effect Linear Regression Analysis for Variation in Overall Cognitive Outcomes, Executive Function and Attention Over Time According to Combined Plasma MCP-1 and Aβ42/40 Status among Community-Dwelling Older Adults**

|  | **Aβ42/40^-a^/MCP1^-b^**  **n=195** | **Aβ42/40^-^ /MCP1^+^**  **n=85** | **Aβ42/40^+^ /MCP1^-^**  **n=97** | **Aβ42/40^+^ /MCP1^+^**  **n=52** |
| --- | --- | --- | --- | --- |
| **Period** | Estimated mean  (95% CI)^c^ | Estimated mean  (95% CI) | Estimated mean  (95% CI) | Estimated mean  (95% CI) |
| **Cognitive Composite Score^d^** | | | | |
| 12 month | -0.09 (-0.12, -0.05) | -0.16 (-0.27, -0.06)* | -0.33 (-0.48, -0.18)*** | -0.32 (-0.51, -0.13)** |
| 24 month | -0.06 (-0.10, -0.03) | -0.14 (-0.25, 0.03) | -0.49 (-0.65, -0.33)*** | -0.34 (-0.54, -0.14)** |
| 36 month | -0.16 (-0.20, -0.12) | -0.28 (-0.39, -0.16) | -0.54 (-0.72, -0.37)*** | -0.38 (-0.60, -0.17)* |
| 48 month | -0.19 (-0.24, -0.15) | -0.32 (-0.45, -0.20) | -0.59 (-0.78, -0.41)*** | -0.60 (-0.83, -0.36)*** |
| **MMSE** | | | | |
| 12 month | -0.12 (-0.26, 0.01) | -0.21 (-0.50, -0.08) | -0.57 (-0.99, -0.16)* | -0.63 (-1.15, -0.10) |
| 24 month | -0.06 (-0.20, 0.08) | -0.27 (-0.58, 0.03) | -1.05 (-1.48, -0.61)*** | -0.52 (-0.97, 0.14) |
| 36 month | -0.28 (-0.43, -0.13) | -0.27 (-0.60, 0.06) | -0.88 (-1.38, -0.38)* | -0.70 (-1.18, -0.06) |
| 48 month | -0.21 (-0.37,-0.05) | -0.34 (-0.69, 0.01) | -0.90 (-1.43, -0.37)* | -0.84 (-1.55, -0.20) |
| **CDR sum of boxes** | | | | |
| 12 month | 0.14 (0.08, 0.20) | 0.16 (0.06, 0.26) | 0.22 (0.04, 0.39) | 0.34 (0.14, 0.53)* |
| 24 month | 0.13 (0.06, 0.20) | 0.22 (0.10, 0.34) | 0.24 (0.03, 0.46) | 0.48 (0.23, 0.73)* |
| 36 month | 0.23 (0.15, 0.31) | 0.26 (0.11, 0.41) | 0.41 (0.13, 0.69) | 0.54 (0.23, 0.85) |
| 48 month | 0.33 (0.23, 0.43) | 0.37 (0.30, 0.54) | 0.75 (0.42, 1.08)* | 0.83 (0.46, 1.20)* |
| **Executive function composite score^e^** | | | | |
| 12 month | -0.02 (-0.05, 0.02) | -0.07 (-0.19, 0.05) | -0.34 (-0.50, -0.17)*** | -0.15 (-0.36, 0.07) |
| 24 month | -0.04 (-0.08, -0.004) | -0.11 (-0.23, 0.01) | -0.39 (-0.56, -0.21)*** | -0.25 (-0.47, -0.02) |
| 36 month | -0.10 (-0.14, -0.05) | -0.17 (-0.30, -0.04) | -0.48 (-0.67, -0.29)*** | -0.33 (-0.58, -0.09) |
| 48 month | -0.12 (-0.17, -0.07) | -0.22 (-0.36, -0.08) | -0.53 (-0.74, -0.33)*** | -0.33 (-0.60, -0.07) |
| **Attention score^f^** | | | | |
| 12 month | -0.03 (-0.05, 0.00) | -0.03 (-0.12, 0.06) | -0.14 (-0.27, -0.01) | -0.16 (-0.32, 0.01) |
| 24 month | -0.03 (-0.06, -0.01) | -0.05 (-0.14, 0.05) | -0.20 (-0.33, -0.06) | -0.25 (-0.41, -0.08)* |
| 36 month | -0.10 (-0.13, -0.07) | -0.14 (-0.24, -0.04) | -0.22 (-0.37, -0.08) | -0.33 (-0.51, -0.15)* |
| 48 month | -0.13 (-0.17, -0.10) | 0.17 (-0.27, -0.07) | -0.32 (-0.48, -0.18)* | -0.42 (-0.61, -0.23)** |

*p-value <0.05; ** p-value <0.001; *** p-value <0.001: Significant differences in the evolution of the outcomes (Aβ42/40^-^/MCP1^-^ as reference group)

^#^p-value <0.05; ^##^ p-value <0.001; ^###^ p-value <0.001: Significant difference in the evolution of the outcomes between Aβ42/40^+^ /MCP1^-^ and Aβ42/40^+^/MCP1^+^ groups.

Models were adjusted by sex, age, BMI, MAPT group, CDR status at baseline, GDS score and ApoE ε4 genotype

Abbreviations: Aβ42/40: β-amyloid 42aa isoform/β-amyloid 40aa isoform ratio; MCP-1: Monocyte Chemoattractant Protein-1; MMSE, Mini-Mental State Examination; CDR, Clinical Dementia Rating; MMSE.

a. Abnormal Aβ42/40 defined as values ≥ 107 pg/mL

b. Abnormal MCP-1 defined as values in the 4th quartile.

c. Negative values indicate worsening performance along follow-up, except for CDR sum of boxes (for which it is given by positive values).

d. Based on the z score of 4 cognitive tests (free and total recall of the Free and Cued Selective Reminding test; 10 MMSE orientation items; Digit Symbol Substitution Test;and Category Naming Test) .

e. Based on the z score of 3 executive function tests (Controlled Oral Word Association Test, the Category Naming Test and the Trail Making Test-Part B)

f. Based on the z score of 2 attention tests (Digit-Symbol Test and the Trail Making Test-Part A)
