# Supplementary material for: Plasma MCP-1 and changes on cognitive function in community-dwelling older adults
Source: Alzheimers Res Ther. 2022 Jan 7;14:5. doi: 10.1186/s13195-021-00940-2 (PMC8742409; doi:10.1186/s13195-021-00940-2)
Supplement: Supplementary file 7 — Additional file 7. Within group evolution in memory outcomes, executive function and attention according to plasma MCP-1 status (excluding ApoE ε4 genotype). Mixed-effect linear regression analysis for variation in memory outcomes over time according to plasma MCP-1 status among community-dwelling older adults (excluding ApoE ε4 genotype). [file 13195_2021_940_MOESM7_ESM.docx]

**Additional File 7. Mixed-Effect Linear Regression Analysis for Variation in Memory Endpoints Over Time According to Combined Plasma MCP-1 and Aβ42/40 Status Among Community-Dwelling Older Adults**

|  | **Aβ42/40^-a^/MCP1^-b^**  **n=195** | **Aβ42/40^-^ /MCP1^+^**  **n=85** | **Aβ42/40^+^ /MCP1^-^**  **n=97** | **Aβ42/40^+^ /MCP1^+^**  **n=52** |
| --- | --- | --- | --- | --- |
| **Period** | Estimated mean  (95% CI)^c^ | Estimated mean  (95% CI) | Estimated mean  (95% CI) | Estimated mean  (95% CI) |
| **FCSRT Free Recall** | | | | |
| 12 month | -1.10 (-1.47, -0.72) | -1.71 (-2.84, -0.58) | -4.36 (-5.96, -2.75) * | -3.84 (-5.85, -1.83)** |
| 24 month | -0.46 (-0.86, -0.06) | -1.40 (-2.58, -0.23) | -4.70 (-6.39, -3.01) *** | -2.82 (-4.96, -0.68)* |
| 36 month | -1.30 (-1.76, -0.84) | -2.89 (-4.16, -1.62)* | -5.76 (-7.64, -3.88) *** | -3.12 (-5.45, -0.78) |
| 48 month | -1.47 (-1.97, -0.98) | -2.98 (-4.32, -1.64)* | -5.69 (-7.71, -3.68) *** | -4.78 (-7.33, -2.23)* |
| **FCSRT Total Recall** | | | | |
| 12 month | -0.60 (-0.84, -0.36) | -0.96 (-1.56, -0.36) | -3.42 (-4.42, -2.42)*** | -2.29 (-3.42, -1.52)*** |
| 24 month | -0.18 (-0.45, 0.08) | -0.53 (-1.17, 0.11) | -3.01 (-4.10, -1.92)*** | -1.89 (-3.15, 0.63)** |
| 36 month | -0.92 (-1.24, -0.61) | -1.23 (-1.94, -0.52) | -3.92 (-5.21, -2.63)*** | -2. 92 (-4.34, 1.50)** |
| 48 month | -0.81 (-1.17, -0.46) | -0.83 (-1.60, -0.06) | -4.75 (-6.19, -3.32)*** | -3.36 (-4.94, -1.77)** |
| **FCSRT Free Delayed Recall** | | | | |
| 12 month | -0.10 (-0.27, 0.06) | -0.35 (-0.80, 0.10) | -1.22 (-1.86, -0.58)*** | -1.59 (-2.39, -0.79)** |
| 24 month | -0.11 (-0.28, 0.07) | -0.38 (-0.85,-0.08) | -1.49 (-2.16, -0.81)*** | -1.25 (-2.11, -0.39)* |
| 36 month | -0.24 (-0.44, -0.04) | -0.84 (-1.36, -0.33)* | -1.61 (-2.38, -0.85)*** | -1.11 (-2.05, -0.16) |
| 48 month | -0.38 (-0.60, -0.17) | -1.07 (-1.61, -0.53)* | -2.28 (-3.10, -1.46)*** | -1.66 (-2.70, -0.62)* |
| **FCSRT Total Delayed Recall** | | | | |
| 12 month | -0.07 (-0.15, 0.01) | -0.29 ( -0.50, -0.09)* | -0.64 (-0.94, -0.34)*** | -0.74 (-1.11, -0.36)*** |
| 24 month | -0.06 (-0.15, 0.03) | -0.38 (-0.61, 0.15)** | -0.78 (-1.13, -0.44)*** | -0.57 (-1.00, -0.13)* |
| 36 month | -0.25 (-0.36, -0.14) | -0.45 (-0.72, -0.18) | -1.25 (-1.66, -0.84)*** | -0.71 (-1.22, -0.19)* |
| 48 month | -0.26 (-0.39 , -0.13) | -0.72 (-1.02, -0.41)** | -1.46 (-1.93, -0.99)*** | -1.19 (-1.79, -0.59)** |

*p-value <0.05; ** p-value <0.001; *** p-value <0.001: Significant differences in the evolution of the outcomes (Aβ42/40^-^/MCP1^-^ as reference group)

^#^p-value <0.05; ^##^ p-value <0.001; ^###^ p-value <0.001: Significant difference in the evolution of the outcomes between Aβ42/40^+^ /MCP1^-^ and Aβ42/40^+^ /MCP1^+^ groups.

Models were adjusted by sex, age, BMI, MAPT group, CDR status at baseline, GDS score and ApoE ε4 genotype

Abbreviations: Aβ42/40: β-amyloid 42aa isoform/β-amyloid 40aa isoform ratio; FCSRT: Free and Cued Selective Reminding Test; MCP-1: Monocyte Chemoattractant Protein-1;

a. Abnormal Aβ42/40 defined as values ≥ 107 pg/mL

b. Abnormal MCP-1 defined as values in the 4th quartile.

c. Negative values indicate worsening performance along follow-up.
