# Supplementary material for: Plasma MCP-1 and changes on cognitive function in community-dwelling older adults
Source: Alzheimers Res Ther. 2022 Jan 7;14:5. doi: 10.1186/s13195-021-00940-2 (PMC8742409; doi:10.1186/s13195-021-00940-2)
Supplement: Supplementary file 8 — Additional file 8. Within group evolution in memory outcomes, executive function and attention according to plasma MCP-1 status (excluding ApoE ε4 genotype). Mixed-effect linear regression analysis for variation in overall cognitive outcomes, executive function and attention over time according to combined plasma Aβ42/40+ and MCP-1 status among community-dwelling older adults (excluding ApoE ε4 genotype). [file 13195_2021_940_MOESM8_ESM.docx]

**Additional File 8. Mixed-Effect Linear Regression Analysis for Variation in Overall Cognitive Outcomes, Executive Function and Attention Over Time According to Combined Plasma MCP-1 and Aβ42/40 Status among Community-Dwelling Older Adults (excluding ApoE ε4 genotype)**

|  | **Aβ42/40^-^/MCP1^-^** | **Aβ42/40^-^ /MCP1^+^** | **Aβ42/40^+^ /MCP1^-^** | **Aβ42/40^+^ /MCP1^+^** |
| --- | --- | --- | --- | --- |
| **Period** | **Estimated mean**  **(95% CI)^c^** | **Estimated mean**  **(95% CI)** | **Estimated mean**  **(95% CI)** | **Estimated mean**  **(95% CI)** |
| **Cognitive Composite Score^d^, n=429** | | | | |
| 12 month | -0.08 (-0.12, -0.05) | -0.05 (-0.16, 0.04) | -0.26 (-0.41, -0.12)*** | -0.19 (-0.37, -0.00)* |
| 24 month | -0.07 (-0.10, -0.03) | -0.05 (-0.16, 0.06) | -0.43 (-0.59, -0.27)*** | -0.24 (-0.44, -0.04)** |
| 36 month | -0.16 (-0.20, -0.12) | -0.09 (-0.21, 0.03) | -0.39 (-0.57, -0.22)*** | -0.20 (-0.42, -0.01) |
| 48 month | -0.20 (-0.24, -0.15) | -0.11 (-0.23, -0.02) | -0.41 (-0.60, -0.22)*** | -0.37 (-0.61, -0.13)** |
| **MMSE, n=429** | | | | |
| 12 month | -0.08 (-0.21, 0.05) | 0.01 (-0.28, 0.29) | -0.49 (-0.91, -0.08)* | -0.43 (-0.96, 0.09) |
| 24 month | -0.07 (-0.20, 0.07) | -0.07 (-0.37, 0.23) | -0.95 (-1.39, -0.51)*** | -0.24 (-0.80, 0.32) |
| 36 month | -0.25 (-0.40, -0.10) | 0.14 (-0.19, 0.48) | -0.60 (-1.12, -0.09)* | -0.20 (-0.82, 0.43) |
| 48 month | -0.19 (-0.34, -0.04) | -0.03 (-0.38, 0.33) | -0.69 (-1.24, -0.14)* | -0.57 (-1.25, 0.12) |
| **CDR sum of boxes, n=429** | | | | |
| 12 month | -0.1 (-0.05, 0.02) | 0.08 (-0.17, 0.06) | 0.06 (-0.10, 0.22) | 0.22 (0.02, 0.42)* |
| 24 month | -0.05 (-0.08, -0.01) | 0.13 (-0.21, 0.02) | 0.10 (-0.10, 0.31) | 0.30 (0.04, 0.55)* |
| 36 month | -0.10 (-0.14, -0.06) | 0.09 (-0.29, -0.04) | 0.19 (-0.07, 0.46) | 0.29 (-0.04, 0.62) |
| 48 month | -0.13 (-0.18, -0.08) | 0.11 (-0.34, -0.07) | 0.35 (0.03, -0.66)* | 0.49 (0.10, 0.88)* |
| **Executive function composite score^e^, n=412** | | | | |
| 12 month | -0.02 (-0.05, 0.02) | -0.04 (-0.15, 0.07) | -0.30 (-0.47, -0.15)*** | -0.05 (-0.26, 0.16) |
| 24 month | -0.04 (-0.08, -0.004) | -0.05 (-0.17, 0.07) | -0.32 (-0.50, -0.17)*** | -0.12 (-0.35, 0.10) |
| 36 month | -0.10 (-0.14, -0.05) | -0.07 (-0.20, -0.06) | -0.37 (-0.56, -0.17)*** | -0.16 (-0.40, 0.08) |
| 48 month | -0.12 (-0.17, -0.07) | -0.07 (-0.21, -0.06) | -0.39 (-0.59, -0.18)*** | -0.12 (-0.39, 0.15) |
| **Attention score^f^, n=429** | | | | |
| 12 month | -0.03 (-0.05, 0.01) | 0.01 (-0.07, 0.10) | -0.10 (-0.23, 0.03) | -0.08 (-0.24, 0.08) |
| 24 month | -0.04 (-0.06, -0.01) | 0.02 (-0.07, 0.10) | -0.15 (-0.29, -0.02)* | -0.18 (-0.35, -0.01)* |
| 36 month | -0.10 (-0.13, -0.07) | 0.02 (-0.08, 0.08) | -0.22 (-0.26, 0.03) | -0.20 (-0.38, -0.03)* |
| 48 month | -0.13 (-0.17, -0.10) | -0.02 (-0.11, -0.06)* | -0.32 (-0.33, -0.03)* | -0.26 (-0.44, -0.07)** |

*p-value <0.05; ** p-value <0.001; *** p-value <0.001: Significant differences in the evolution of the outcomes (Aβ42/40^-^/MCP1^-^ as reference group)

^#^p-value <0.05; ^##^ p-value <0.001; ^###^ p-value <0.001: Significant difference in the evolution of the outcomes between Aβ42/40^+^ /MCP1^-^ and Aβ42/40^+^/MCP1^+^ groups.

Models were adjusted by sex, age, BMI, MAPT group, CDR status at baseline, GDS score and ApoE ε4 genotypeAbbreviations: Aβ42/40: β-amyloid 42aa isoform/β-amyloid 40aa isoform ratio; MCP-1: Monocyte Chemoattractant Protein-1; MMSE, Mini-Mental State Examination; CDR, Clinical Dementia Rating; MMSE.

a. Abnormal Aβ42/40 defined as values ≥ 107 pg/mL

b. Abnormal MCP-1 defined as values in the 4th quartile.

c. Negative values indicate worsening performance along follow-up, except for CDR sum of boxes (for which it is given by positive values).

d. Based on the z score of 4 cognitive tests (free and total recall of the Free and Cued Selective Reminding test; 10 MMSE orientation items; Digit Symbol Substitution Test;and Category Naming Test) .

e. Based on the z score of 3 executive function tests (Controlled Oral Word Association Test, the Category Naming Test and the Trail Making Test-Part B)

f. Based on the z score of 2 attention tests (Digit-Symbol Test and the Trail Making Test-Part A)
