# Supplementary material for: Plasma MCP-1 and changes on cognitive function in community-dwelling older adults
Source: Alzheimers Res Ther. 2022 Jan 7;14:5. doi: 10.1186/s13195-021-00940-2 (PMC8742409; doi:10.1186/s13195-021-00940-2)
Supplement: Supplementary file 9 — Additional file 9. Within group evolution in memory outcomes, executive function and attention according to plasma MCP-1 status (excluding ApoE ε4 genotype). Mixed-effect linear regression analysis for variation in memory outcomes over time according to combined plasma Aβ42/40+ and MCP-1 status among community-dwelling older adults (excluding ApoE ε4 genotype). [file 13195_2021_940_MOESM9_ESM.docx]

**Additional File 9. Mixed-Effect Linear Regression Analysis for Variation in Memory Outcomes Over Time According to Combined Plasma MCP-1 and Aβ42/40 Status Among Community-Dwelling Older Adults (excluding ApoE ε4 genotype)**

|  | **Aβ42/40^-a^/MCP1^-b^**  **n=195** | **Aβ42/40^-^ /MCP1^+^**  **n=85** | **Aβ42/40^+^ /MCP1^-^**  **n=97** | **Aβ42/40^+^ /MCP1^+^**  **n=52** |
| --- | --- | --- | --- | --- |
| **Period** | Estimated mean  (95% CI)^c^ | Estimated mean  (95% CI) | Estimated mean  (95% CI) | Estimated mean  (95% CI) |
| **FCSRT Free Recall, n=429** | | | | |
| 12 month | -1.05 (-1.43, -0.69) | -0.67 (-1.76, 0.42) | -3.36 (-4.96, -1.76)*** | -2.10 (-4.09, -0.12)** |
| 24 month | -0.48 (-0.87, -0.10) | -0.84 (-1.98, 0.31) | -4.26 (-5.96, -2.57)*** | -2.05 (-4.17, 0.08) |
| 36 month | -1.26 (-1.70, -0.82) | -1.58 (-2.84, -0.32)* | -4.55 (-6.45, -3.93)*** | -1.86 (-4.18, 0.46) |
| 48 month | -1.48 (-1.95, -0.99) | -1.50 (-2.84, -0.15)* | -4.27 (-6.32, -3.74)*** | -2.91 (-5.44, -0.37)* |
| **FCSRT Total Recall, n=429** | | | | |
| 12 month | -0.62 (-0.85, -0.40) | -0.68 (-1.33, -0.04)* | -2.63 (-3.58, -1.67)*** | -2.77 (-3.97, -1.56) |
| 24 month | -0.27 (-0.51, -0.03) | -0.48 (-1.19, 0.21) | -2.56 (-3.62, -1.51)*** | -3.74 (-2.78, -1.06)* |
| 36 month | -1.02 (-1.31, -0.73) | -0.43 (-1.23, 0.37) | -2.65 (-3.88, -1.43)*** | -4.89 (-4.34, -1.84)* |
| 48 month | -0.92 (-1.25, -0.60) | -0.17 (-1.05, 0.72) | -3.44 (-4.81, -2.08)*** | -3.75 (-5.47, -2.03)** |
| **FCSRT Free Delayed Recall, n=429** | | | | |
| 12 month | **-0.10 (-0.26, 0.06)** | -0.28 (-0.71, 0.16) | -1.27 (-1.91, -0.63)*** | -1.36 (-2.16, -0.57)*** |
| 24 month | -0.13 (-0.30, 0.04) | -0.34 (-0.79, 0.11) | -1.52 (-2.21, -0.84)*** | -1.14 (-1.99, -0.28)** |
| 36 month | -0.24 (-0.43, -0.05) | -0.80 (-1.29, -0.30)* | -1.66 (-2.42, -0.89)*** | -1.05 (-1.98, -0.10) |
| 48 month | -0.39 (-0.60, -0.18) | -1.04 (-1.57, -0.52)* | -2.33 (-3.15, -1.50)*** | -1.62 (-2.65, -0.59)* |
| **FCSRT Total Delayed Recall, n=429** | | | | |
| 12 month | -0.08 (-0.16, -0.004) | -0.10 (-0.32, 0.11) | -0.68 (-0.99, -0.37)** | -0.54 (-0.93, -0.15)** |
| 24 month | -0.07 (-0.16, 0.02) | -0.21 (-0.45, 0.03) | -0.79 (-1.15, -0.43)** | -0.39 (-0.84, 0.06) |
| 36 month | -0.24 (-0.34, -0.13) | -0.10 (-0.39, 0.18) | -1.09 (-1.53, -0.66)*** | -0.35 (-0.88, 0.18) |
| 48 month | -0.27 (-0.39, -0.15) | -0.38 (-0.70, -0.06)* | -1.28 (-1.77, -0.79)*** | -0.81 (-1.42, -0.20)** |

*p-value <0.05; ** p-value <0.001; *** p-value <0.001: Significant differences in the evolution of the outcomes (Aβ42/40^-^/MCP1^-^ as reference group)

^#^p-value <0.05; ^##^ p-value <0.001; ^###^ p-value <0.001: Significant difference in the evolution of the outcomes between Aβ42/40^+^ /MCP1^-^ and Aβ42/40^+^/MCP1^+^ groups.

Models were adjusted by sex, age, BMI, MAPT group, CDR status at baseline, GDS score and ApoE ε4 genotype

Abbreviations: Aβ42/40: β-amyloid 42aa isoform/β-amyloid 40aa isoform ratio; MCP-1: Monocyte Chemoattractant Protein-1; MMSE, Mini-Mental State Examination; CDR, Clinical Dementia Rating; MMSE.

a. Abnormal Aβ42/40 defined as values ≥ 107 pg/mL

b. Abnormal MCP-1 defined as values in the 4th quartile.

c. Negative values indicate worsening performance along follow-up, except for CDR sum of boxes (for which it is given by positive values).

d. Based on the z score of 4 cognitive tests (free and total recall of the Free and Cued Selective Reminding test; 10 MMSE orientation items; Digit Symbol Substitution Test;and Category Naming Test) .

e. Based on the z score of 3 executive function tests (Controlled Oral Word Association Test, the Category Naming Test and the Trail Making Test-Part B)

f. Based on the z score of 2 attention tests (Digit-Symbol Test and the Trail Making Test-Part A)
